# Targeting of small molecule anticancer drugs to the tumour and its vasculature using cationic liposomes: lessons from gene therapy

**DOI:** 10.1186/1475-2867-6-17

**Published:** 2006-06-23

**Authors:** Crispin R Dass, Peter FM Choong

**Affiliations:** 1 Department of Orthopaedics, University of Melbourne, St. Vincent's Hospital Melbourne, Australia; 2 Bone and Soft Tissue Sarcoma Service, Peter MacCallum Cancer Institute, Melbourne, Australia

## Abstract

Cationic (positively charged) liposomes have been tested in various gene therapy clinical trials for neoplastic and other diseases. They have demonstrated selectivity for tumour vascular endothelial cells raising hopes for both antiangiogenic and antivascular therapies. They are also capable of being selectively delivered to the lungs and liver when administered intravenously. These vesicles are being targeted to the tumour in various parts of the body by using advanced liposomal systems such as ligand-receptor and antibody-antigen combinations. At present, the transferrin receptor is commonly used for cancer-targeted drug delivery systems including cationic liposomes. This review looks at the growing utility of these vesicles for delivery of small molecule anticancer drugs.

## Introduction

Modern medicine is successful in achieving disease-free survival in a good number of cancer patients. However, in a majority of cases, medical intervention is only successful in prolonging the life of a patient from months to a few years. Cancer is essentially a pathology with various mechanisms at its disposal to avert its own destruction. Thus, multi-modal therapy is required, with or without surgical intervention. Novel therapies are constantly being discovered, developed and trialled, with neoplasia targeting given high priority. For all forms of therapies, a common thread is the need for targeting to avoid side-effects of drugs. In the past few years, cationic liposomes (CLs) have been shown to be selective for tumour vascular endothelial cells (VECs). In addition, as mentioned below, a handful of papers highlight the ability of targeting these vesicles to tumours in various parts of the body by using advanced liposome drug delivery systems (DDSs).

Molecular therapy, is a promising strategy for the treatment of human disease. However, as above-mentioned, delivery of molecular therapeutics efficiently and specifically to the target tissue remains a significant challenge. A great deal of research has been done to address the difficulties faced by a drug molecule as it leaves its site of administration and distributes in the body, hopefully reaching the target site at doses sufficient to alter the pathology, or halt disease progression. Whilst this is true generically for all diseases amenable to drug therapy, it is particularly so for cancer, which challenges modern medicine with the ability to grow at a rapid rate, actively pump out drugs from cancer cells, and spread through the bloodstream to reside in secondary spots in the body.

It is the process of metastasis (cancer spread), which eventually kills the patient in most cases. Current surgery can quite aptly remove much of the bulk of a primary tumour and is useful in certain types of metastectomy, such as in the lungs of osteosarcoma patients. Radiotherapy is utilised in specific types of tumours, but chemotherapy is very common for a variety of cancers. Regimes using multiple drugs for the one patient is more the norm than the exception nowadays. These conventional forms of therapies do have success in quite a lot of patients, but there is still that gap in knowledge as to what causes a cancer to initiate, become latent, then progress rapidly, and finally spread. Such answers can only come about with increased basic academic research. Nevertheless, one of the promising offshoots of basic research has been the emergence of novel forms of therapies for cancer or the re-examination of various ways to better target diseased tissue.

For cancer, regional or local administration is possible for certain lesions such as head and neck cancer and melanoma. For deep-seated tumours, limited targeting has been achieved via selective delivery using upstream intraarterial administration of microspheres [1], use of immunoliposomes [2], and exploitation of ligand-receptor interactions [3], mostly at the preclinical stage. The ability of the above examples has been extensively demonstrated *in vitro *with cultured cells. However, there is a paucity of literature following up with *in vivo *demonstration for a majority of them.

Thus, the main hurdle is how to fulfil the targeting aspect of these concepts when treating a tumour in an organism. In general, only those ideas that are realised by the merging of different concepts seem to proceed and deliver results, albeit hardly impressively. Thus, there is a real need for better ways to deliver therapeutics to tumours *in vivo*. This review looks at these issues and ways to improve on the current situation for CLs, an entity that has attributes placing it among one of the most promising delivery agents for targeting small molecule therapy for cancer. For the purposes of this review, small molecule drugs refer to cytotoxic agents which are xenobiotic and which are much smaller chemically than biologicals such as protein or oligonucleotides.

## Cationic liposomes

It is nearly two decades now since a cationic lipid was seminally used to introduce plasmid DNA into cells [4]. Since then, numerous cationic liposomes (CLs, also called cytofectins or lipofection reagents) have been synthesised and used for delivery of nucleic acids into cells in culture, in animals and in patients enrolled in phase I and II clinical trials. In comparison to other gene delivery modes, such as viral vectors, CLs, the most common transfection reagent, are technically simple and quick to formulate, are not as biologically hazardous as viral vectors, are readily available commercially, and may be tailored for specific applications.

After the initial surge in use of CLs for transfection of cultured cells and gene transfer in animals, the realisation that they had limitations, prompted a re-evaluation of their design. The overriding concern was the degree of toxicity that CLs exhibited in cultured cells and that these effects were at times drastically pronounced in several animal studies [5]. When low doses of CLs are employed *in vivo*, transfection results are only slightly better than naked gene delivery, thus signalling the need for administration of higher doses, which then tend to be toxic. In light of the fine balance between toxicity and efficacy, the past seven years has brought about a major re-emphasis on vehicle safety.

Recently, there has been renewed interest in cationic liposomes, mainly due to their inherent yet unexplained ability to target certain features of a growing tumour mass. These vesicles have been shown, as specific examples below will highlight, the ability not only to target carried agents to the tumour cells, but the suppliant vasculature endothelial cells, thereby having great utility in anti-angiogenesis and anti-vascular therapy.

The role of non-cationic helper lipids such as the neutral dioleoylphosphatidylethanolamine (DOPE) is to facilitate membrane fusion and aid in the destabilisation of the plasmalemma or endosome [6]. In addition, these supporting lipids stabilise the cationic liposome suspension as cationic lipids repel each other [7] and to counteract the uptake-opposing effects of anionic glycosaminoglycans noted in other carriers such as polyethyleneimine (PEI) and dendrimers [8]. Liposomes formulated without neutral lipid(s) have inferior rates of cellular uptake [9], whilst varying rates may result from varying ratios of cationic:neutral lipid used to formulate the liposomes [10,11].

As above-mentioned, the success of cationic liposome-mediated nucleic acid transfer is dependent on numerous factors that may explain the inherent variability of lipofection (lipoplex-mediated transfection), particularly *in vivo *[1,12]. These vehicles have been proven to be non-toxic in a majority of investigations, including phase I and II clinical trials, albeit varying degrees of toxicity still emerge occasionally [reviewed in 13]. Some of this is due to the carried nucleic acid, whilst others are due to the cationic lipidic components of the vesicle, or in fact even the combined effects of the lipoplexes formed.

Some of the earlier generation cationic lipids such as DMRIE [()-N-(2-hydroxyethyl)-N, N-dimethyl-2, 3-bis(tetradecycloxy)-1-propanaminium bromide] and DC-Chol were tested in clinical trials [reviewed in 13], but the resultant biological (therapeutic) effects with these vesicles were at best marginal, with toxicity overshadowing any beneficial effects of transgene expression. Recent research has pinpointed certain features of CLs that enhance their capability for nucleic acid transport *in vivo*. These may also be highly relevant to small molecule delivery and include the cationic head group and its neighbouring aliphatic chain being in a 1,2-relationship on the backbone, an ether bond for bridging the aliphatic chains to the backbone, and paired oleyl chains acting as the hydrophobic tether [14]. Ester bonds within the linker region are believed to be better due to their degradation in cells, thereby reducing cytotoxicity [15]. Biodegradation is currently a key feature sought in DDSs.

In any case, these features, whilst not determining better transfection capacity in cell culture, facilitate better nucleic acid delivery *in vivo*. Thus, *in vitro *and cell culture results have to be treated with caution and cannot necessarily be used to extrapolate the genuine potential of a nucleic acid carrier *in vivo*. Other factors such as particle diameter and route of administration become more important when these vesicles are introduced *in vivo *[16]. Finally, what may be a good lipofection reagent for one application, may not necessarily prove to be ideal for another. A substantial quantity of empirical research to determine the optimal conditions for *in vitro *and *in vivo *transfection with CLs is usually required.

## Evidence for selective delivery of CLs to tumour VECs

It is now a well-known fact that CLs target quite selectively, the vasculature of tumours, a phenomenon not noted with anionic (negatively charged) or electroneutral (zero charge potential) liposomes [17]. Campbell and colleagues [18] found that CLs, sterically stabilised with the addition of 5% molar PEG, accumulated more in such vessels when CLs were used as opposed to neutral charged liposomes. Inclusion of PEGylated lipids in the vesicles has the added advantage of reducing aggregate formation [19], thus increasing both yield and injectability of complexes. Unmodified lipoplexes have a relatively short circulation half-life of less than 5 minutes [18]. Furthermore, when the percentage of cationic lipid is increased from 10 to 50% molar, the accumulation in tumour VECs increases by 100%.

The inclusion of PEGylated lipids delay liposome clearance from blood, but not at the expense of interaction and uptake by tumour VECs [18]. It is a well-known fact that the inclusion of PEGylated lipids on the liposome surface significantly increases circulation half-life *in vivo*. In contrast, no change in interstitial accumulation could be detected. This selective delivery to tumour VECs was noted in two human tumour types (LS174T and MCAIV) and at two locations (cranial window and dorsal skin fold chamber). Distribution of vesicles in tumour vessels was heterogeneous, and this may have some bearing on whether this technology is powerful enough to destroy enough tumour VECs to induce tumour regression. Interestingly, a 50% molar charge on the liposomes significantly increased accumulation in the lungs of mice 24 h post-injection. Thus, for pulmonary metastasis, this could be of substantial benefit.

Based on several lines of evidence, it is believed that mammalian cells interact with and internalise cationic macromolecules by endocytosis [20], and that this interaction is at least partially mediated by proteoglycans [21]. Furthermore, in the case of mosaic tumour vessels (vessels comprised of both VECs and tumour cells), the tumour cells are in direct contact with cationised macromolecules, including CLs, and uptake should occur in both VECs and neoplastic cells [22]. Thus, in theory at least, cargo delivered by CLs should selectively be delivered to the supporting neovasculature and directly to tumour cells. Mitotic index is believed to play a major role in the uptake of delivered agents selectively to tumour cells [23].

Earlier on, Thurston and coworkers [24] demonstrated that in the RIP-Tag2 and the K14-HPV16 tumour models, the quantity of liposomes accumulating in tumour vessels were up to 33-fold than that in corresponding vessels in non-tumour-bearing mice. Of the CLs inside tumour VECs, 89% were in multivesicular bodies, 10% in small vesicles, and ~1% in complex structures composed of multiple interconnecting vesicles, all probably at various stages of the vesiculo-vacuolar organelle (VVO) system.

Notable was the observation that within 20 minutes of intravenous injection, liposomes appeared on the luminal surface of angiogenic VECs or in vesicular structures within these cells [24]. Furthermore, 51% of the CLs accumulating on the tumour VEC surface were associated with fenestrae, although fenestrae constituted a mere 4% of the luminal VEC surface. This could be attributed to the positive charge on the vesicle surface, since cationic ferritin, but not native ferritin, exhibits ready binding to fenestrae, suggesting the presence of anionic moieties on fenestral diaphragms [25]. Such interaction may take place at the coated pit present on VECs, since these sites are known to bind cationic molecules, albeit guiding them down the endosomal pathway to destruction [26]. Thurston and colleagues [24] put forward several lines of evidence indicating that extravasation was due to trans-VEC transport rather than *via *leakage through the basement membranes of tumour endothelium.

## Selective delivery of small molecule drugs to tumour and its vasculature

The vascular network is naturally highly accessible to intravascularly-administered therapeutic agents. Regardless of the route of administration, once the agent gains access to the circulatory system, it has the potential to target actively proliferating vessels, such as those in a tumour. The vasculature of the tumour also occupies a relatively small area in comparison to the tumour interstitium, thus the doses of antiangiogenic agents to be delivered *in vivo *should theoretically be much less than what needs to be administered for general anticancer chemotherapeutics.

Conventional cytotoxic agents that recently have been found to be antiangiogenic, such as vinblastine and paclitaxel, would need to be injected in much smaller doses, albeit maybe more frequently in keeping with metronomic dosing schedules [27]. Regardless of efficacy, the effects of such targeted therapy has to be monitored in models also looking at whether physiological angiogenesis, such as that occurring in the menstrual cycle and wound healing, is perturbed as well.

CLs are capable of causing an antivascular effect with cytotoxic agents. For instance, Kunstfeld and coworkers [28] demonstrated that paclitaxel encapsulated in CLs diminishes tumour angiogenesis and inhibits orthotopic melanoma growth in SCID mice. In contrast, paclitaxel administered in its normal Cremophor EL medium, while showing an inhibitory effect in cell culture, was unable to significantly decrease angiogenesis and tumour growth *in vivo*. Kunstfeld and colleagues [28] speculate further that factors governing constitutive mitosis, such as that in normal physiological processes, are different from that inducing division in angiogenic vessels.

Strieth and colleagues [29] used the dorsal skinfold chamber method, A-Mel-3 melanomas and intravenous paclitaxel to demonstrate that tumour growth was significantly inhibited after treatment with paclitaxel within CLs compared to the treatment with non-liposomal paclitaxel. Encapsulated paclitaxel caused a decrease of functional tumour vessel density and a constriction of vessel diameters. This resulted in a significantly reduced blood flow in vessel segments and a reduced microcirculatory perfusion index in these animals. The degree of apoptosis in the vicinity of the vessels was significantly increased when animals were administered liposomal paclitaxel.

Schmitt-Sody and colleagues [30], in A-Mel-3 tumours in dorsal skinfold preparations, demonstrated that vascular targeting of paclitaxel-containing CLs was achieved after encapsulation. Tumour growth was significantly inhibited when compared to control groups. In addition, the appearance of regional lymph node metastases was significantly delayed by treatment with paclitaxel encapsulated into CLs in comparison with all other groups. Krasnici and coworkers [31] utilised A-Mel-3 growing in the dorsal skinfold to demonstrate that after intravenous application of anionic and neutral liposomes, there was no specific targeting to tumour tissue. In contrast, CLs exhibited a significantly enhanced accumulation in tumour tissue and tumour vasculature up to 3-fold compared to surrounding tissue within 20 minutes post-administration.

**Figure 1:**
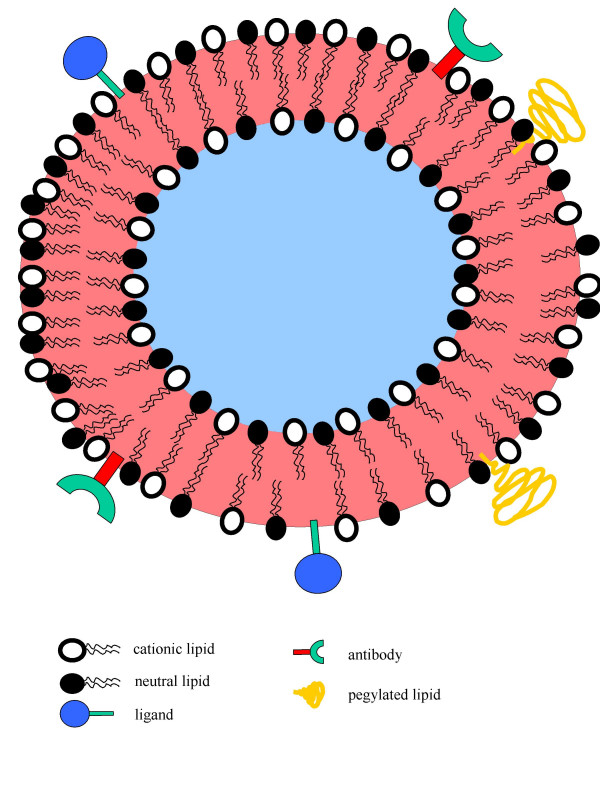
An ideal cationic liposome. A vesicle capable of targeting tumour vessels and tumour cells *in vivo *and that consists of two compartments – hydrophilic core (blue) and hydrophobic sandwiched bilayer (red).

Thus, there is ample evidence that CLs have the inherent potential for selective delivery to tumour VECs. Surprisingly, not much more work has been done to exploit this phenomenon. However, the results of studies [28-30] have led to the phase I trial of the first such complex termed MBT-0206, which is comprised of Taxol^® ^and the lipidic vehicle called EndoTAG™. Nevertheless, CLs have also been shown to target other tissues in the body, albeit with substantial modifications to their overall structure as highlighted in the examples below. As shown in Figure 1, further modifications to CLs may indeed increase their cancer targeting ability. Such modifications include inclusion of pegylated lipids for enhanced circulation. Tumour cell specific ligands or antibodies may be included on the liposome coat to allow specificity of drug delivery. Finally, because these liposomes can be formulated with bilayers, they may ferry both hydrophobic drugs (between the lipid bilayer sandwich) and hydrophilic drugs (within the aqueous space in the core of the vesicles). In addition, their positive charge-coated surface may also carry anionic drugs purely by electrostatic attraction.

## Targeted liposomal delivery of transgenes to non-endothelium tissues

CLs have been used to selectively deliver their genetic payload to tissues other than the endothelium. As the specific examples below show, most work has been carried out with either ligand-receptor or antibody-antigen recognition. The increasing number of papers detailing *in vivo *success with these entities especially for gene transfer attest to the latent potential of this technology for targeting small molecular cytotoxics as well. A common system used is that of the transferrin (Tf) receptor (TfR) for binding and cell entry, as these TfRs are over-expressed by a variety of tumour cells and are widely being investigated for tumour-targeted drug delivery. Many neoplastic cells overexpress the transferrin receptor to increase their iron uptake.

The use of the Tf ligand to target a CL delivery system resulted in a significant increase in the transfection efficiency of the complex [32]. Delivery of wild-type (wt) p53 to a radiation-resistant squamous cell carcinoma of the head and neck (SCCHN) cell line *via *this ligand-targeted, liposome complex was also able to revert the radiation resistant phenotype of these cells *in vitro*. The Tf-targeted CL-DNA complex showed high gene transfer efficiency and efficacy with human head and neck cancer *in vitro *and *in vivo *[33]. Intravenous liposome administration of wt p53 markedly sensitized established SCCHN nude mouse xenograft tumors to radiotherapy, and led to complete tumour regression. It is not hard to imagine that a similar ligand-targeted approach can be highly efficacious for small molecule therapy.

Xu and colleagues [34] then went on to describe a novel cationic immunolipoplex system that showed high *in vivo *gene transfer efficiency and anti- tumour efficacy when used for systemic p53 gene therapy of cancer. The novel cationic immunolipoplex incorporating a biosynthetically lipid-tagged, anti-transferrin receptor single-chain antibody (TfRscFv) targeted tumour cells both *in vitro *and *in vivo*. A human breast cancer metastasis model was employed to demonstrate that the TfRscFv-targeting cationic immunolipoplex enhanced tumour cell binding, and improved targeted gene delivery and transfection efficiencies. The combination of the p53 gene delivered by the systemic administration of the TfRscFv-immunolipoplex and docetaxel resulted in significantly improved efficacy with prolonged survival.

Xu and coworkers [35] explored the structure, size, formation process, and structure-function relationships of a Tf-lipoplex. They observed that Tf-lipoplex had a highly compact structure, with a relatively uniform size of 50–90 nm, resembling a virus particle with a dense core enveloped by a membrane coated with Tf molecules spiking the surface. The Tf-lipoplex showed enhanced stability, improved *in vivo *gene transfer efficiency, and long-term efficacy for systemic p53 gene therapy of human prostate cancer when used in combination with conventional radiotherapy. A multistep self-assembly process and a Tf-facilitated DNA co-condensation model that may provide an explanation for the resultant small size and effectiveness of the nanostructural Tf-lipoplex system was proposed by the authors. For small molecules, reducing particle size will be easier since these chemicals structures are significantly smaller and lack even secondary structure in comparison to long DNA or RNA strands.

TfRscFv has a number of advantages over the transferrin (Tf) molecule itself, including the fact that scFv has a much smaller size than Tf producing a smaller immunolipoplex giving better penetration into solid tumors. Unlike Tf, scFv is a recombinant protein, not a blood product, and thus large scale production and strict quality control of the recombinant scFv, as well as scFv-immunolipoplex, are possible. The sensitization of tumours to chemotherapy by this tumour-targeted and efficient gene transfer method could lower the effective dose of the drug, correspondingly lessening the severe side effects, while decreasing the possibility of recurrence. Moreover, this approach is applicable to both primary and recurrent tumours, and more significantly, metastatic disease. However, one area of improvement is to increase the low yield of this lipid-tagged scFv to facilitate further development and studies.

Yu and colleagues [36] developed a sterically stabilized immunolipoplex (TsPLP), containing an antitransferrin receptor single-chain antibody fragment (TfRscFv)-PEG molecule, to specifically and efficiently deliver a therapeutic gene to tumour cells. The lipoplex was formed first and then sequentially conjugated with PEG and TfRscFv. The complex prepared by this method was shown to be superior in ability to target genes to tumour cells than when prepared by a common precoating strategy, in which DNA is mixed with TfRscFv-PEG conjugated liposome. Using the prostate cancer cell line DU145, it was found that the level of exogenous gene expression in the TSPLP transfected tumours was 2-fold higher than non-PEGylated liposomes and transgene expression did not decrease over time. More importantly, high exogenous gene expression in tumour, but low expression in liver, was observed after an intravenous delivery of TsPLP.

The above technology has been shown to be efficient for targeting plasmid, antisense and siRNA strands to tumours *in vivo *[37]. Thus, since targeting is to a naturally present receptor system on cells that is overrepresented in cancer cells, in essence, targeting of any biomolecule that is able to be linked to the ligand of the receptor or an antibody fragment to it should theoretically facilitate delivery to tumours selectively *in vivo*. Use of CLs with PEGylated lipids and TfR-targeted moieties not only should target tumour tissue, but enhance circulation time and also be capable of selectively destroying tumour VECs. This dual-pronged effect makes this mechanism a highly attractive one and warrants further development.

## Foreseeable problems for small molecule delivery using CLs

While cationic agents may be effective at mediating DNA uptake in cells in culture or when delivered locally *in vivo*, their use as systemic delivery agents is limited due to the size and high superficial charge of the lipid complexes. Thus, controlled release systems with minimal charge and optimum particle diameter are needed. This entails a lot of empirical research at the pre-*in vivo *stage. For the former, optimisation of positive-to-negative charge ratio is important, while for the latter, sizing variations using common techniques such as extrusion, freeze-thaw, and sonication need to be empirically determined.

For lipoplexes, intravenous delivery of oligonucleotides complexed with DC-Chol/DOPE in mice leads to a rapid deposition in the capillary beds of the lung followed by release into the plasma and ultimate clearance into the spleen and liver [38]. This may not be the case for small molecule drugs as they theoretically should not form such complexes due to their small chemical structure and significantly less charge per molecule. Oligonucleotides, being highly anionic, are notorious for forming bridging bonds between complexes, forming complexes that even becoming macroscopic and sometimes precipitating out of solution [39].

On the other hand, some complexation may prove to be beneficial. The natural tendency for lung and liver uptake *via *intravenous administration may be exploited for eradication of pulmonary and hepatic metastases. Such an approach has been taken by several groups with varying degrees of success for pulmonary [40,41] and hepatic [42,43] metastases gene delivery. The inclusion of additional molecules into liposomes that could cause aggregation, such as oligonucleotides, may abet such delivery to metastatic growths. However, it would be important to predetermine whether such additions do not interfere with drug-target interactions at the diseased site. The challenge, once again, is to limit distribution of therapeutics to other healthy tissues.

## Future directions

The fact that CLs are able to selectively deliver their carried load to certain tissues such as tumour endothelium, lungs and liver, make them quite attractive commodities for cancer therapy. However, on the downside, these vesicles tend to aggregate and have the potential to form microemboli *in vivo*. Tissue ischaemia may be problematic, but in the case of tumours, shutting down of the blood supply is in fact very much a goal. In any case, these microemboli may then serve as depots from which a sustained release of lipoplexes from within the tumour microvascular bed may occur. Such controlled release delivery is a highly desired feature in any therapeutic DDS and has been shown to work with microspheres [1,44], even clinically [45,46]. This system of selective delivery is particularly useful for osteosarcoma and renal cancers [47], where the vasculature is well established.

New techniques such as those used for preparing coated CLs [48], have shown promise *in vivo *for targeting to hepatic VECs when liposomal surfaces were embedded with human serum albumin [49]. Docking of liposomes on to microspheres has also been shown to enhance tumour:normal gene delivery *in vivo *when administered upstream of the tumour intraarterially [50]. Furthermore, it has recently been shown that coating adenoviral gene vectors with CLs shields them from immunorecognition of these novel entities, albeit at the expense of a higher rate of cytotoxicity [51].

Stabilised lipid particles [52], containing a PEGylated lipid for extended circulation of what is essentially a CL, has recently been shown to bypass so-called 'first pass' organs, including the lung, and elicit levels of gene expression in distal tumour tissue 100- to 1000-fold greater than that observed in normal tissues [53]. These vesicles load the plasmids using the positive charge of the cationic lipidic constituent of the vesicle, before the charge is neutralised so that *in vivo *both cytotoxicity and opsonisation may be reduced. One criticism of such a modification may be whether the inherent anticancer properties of the cationic lipids [13] are reduced when the charge of these lipids are masked.

For most types of liposomes, the field has had to evolve and use a variety of methods incorporating the positive aspects of different vesicles to create better new generation DDSs (Figure 1) capable of ferrying both gene vectors as well as small molecule drugs such as paclitaxel. This has been the case at least for the past decade [54] and it is apparent that such a challenge will take significant time and substantial resources before significant improvements over the existing liposomes take place. Amongst all the variety of liposomes, CLs have been one class that have been developed, tested and modified both rapidly and intensively.

In fact, one may argue that CLs have given liposomologists a much needed boost in their efforts to enhance drug pharmacokinetics. These vesicles were taken into the clinic within a decade of their discovery, an amazing feat *per se*. Clinical evaluation was preceded by numerous studies looking at the safety, pharmacokinetics and pharmacodynamics in animals. Even when faced with mounting criticism, CLs have persisted and as yet have to be replaced by other types of liposomes or other types of gene delivery mechanisms such as viral vectors.

Further fine-tuning is definitely required, but with the quantity and quality of research being carried out, the task does not seem insurmountable anymore. The search for elements for fine-tuning of CLs should not be limited to lipidic or non-lipidic components, but other aspects of drug delivery such as choosing better routes of administration. The past of focussed research in well-defined, generally mutually exclusive circles have well and truly been replaced with research where delineations are blurred and the central aim is to help those suffering from ailments in the most rapid format that is possible.

## Summary

Cationic liposomes, which have been around for 2 decades now, have been tested in various gene therapy clinical trials for cancer and other genetic pathologies. These class of vesicles have selectivity for tumour vascular endothelial cells as well as being selectively delivered to the lungs and liver when administered intravenously. These vesicles are capable of targeting the tumour *via *advanced liposomal systems such as antibody-antigen or ligand-receptor recognition. While delivery of genes and gene-modulating oligonucleotides are the norm for this class of vehicles, they are increasingly being tested for conventionally drug delivery as well. Fine-tuning is needed if small molecule drug delivery with these carriers are to be adopted for clinical testing.

## Abbreviations

CL, cationic liposome, DDS, drug delivery system, Tf, transferrin, TfR, Tf receptor, VEC, vascular endothelial cell
